# Toward the development of transcriptional biodosimetry for the identification of irradiated individuals and assessment of absorbed radiation dose

**DOI:** 10.1007/s00411-015-0603-8

**Published:** 2015-05-14

**Authors:** Kamil Brzóska, Marcin Kruszewski

**Affiliations:** Centre for Radiobiology and Biological Dosimetry, Institute of Nuclear Chemistry and Technology, Dorodna 16, 03-195 Warsaw, Poland; Department of Medical Biology and Translational Research, Faculty of Medicine, University of Information Technology and Management, Sucharskiego 2, 35-225 Rzeszów, Poland

**Keywords:** Biological dosimetry, Gene expression, qPCR, Transcriptional biomarkers

## Abstract

The most frequently used and the best established method of biological dosimetry at present is the dicentric chromosome assay, which is poorly suitable for a mass casualties scenario. This gives rise to the need for the development of new, high-throughput assays for rapid identification of the subjects exposed to ionizing radiation. In the present study, we tested the usefulness of gene expression analysis in blood cells for biological dosimetry. Human peripheral blood from three healthy donors was X-irradiated with doses of 0 (control), 0.6, and 2 Gy. The mRNA level of 16 genes (*ATF3*, *BAX*, *BBC3*, *BCL2*, *CDKN1A*, *DDB2*, *FDXR*, *GADD45A*, *GDF15*, *MDM2*, *PLK3*, *SERPINE1*, *SESN2*, *TNFRSF10B*, *TNFSF4*, and *VWCE*) was assessed by reverse transcription quantitative PCR 6, 12, 24, and 48 h after exposure with *ITFG1* and *DPM1* used as a reference genes. The panel of radiation-responsive genes was selected comprising *GADD45A*, *CDKN1A*, *BAX*, *BBC3*, *DDB2*, *TNFSF4*, *GDF15*, and *FDXR*. Cluster analysis showed that Δ*C*_t_ values of the selected genes contained sufficient information to allow discrimination between irradiated and non-irradiated blood samples. The samples were clearly grouped according to the absorbed doses of radiation and not to the time interval after irradiation or to the blood donor.

**Electronic supplementary material:**

The online version of this article (doi:10.1007/s00411-015-0603-8) contains supplementary material, which is available to authorized users.

## Introduction

Biological dosimetry is the quantification of exposure to ionizing radiation by means of measurable biological changes (biological indicators) that take place in the biological system. Based on such indicators, cases of individual exposure to ionizing radiation can be detected and possible consequences of the exposure predicted. This enables the planning of adequate medical treatment, when information from physical dosimetry is not available (Stephan et al. 2007).

Currently, the most frequently used and the best established method of biological dosimetry is the dicentric chromosome assay (DCA). It has many advantages, such as high specificity for ionizing radiation and low background in the healthy general population (about 1–2 dicentrics per 1000 cells) (Pinto et al. 2010). Although dicentric chromosomes are unstable and cells bearing such aberrations are eliminated from the circulating lymphocyte pool, the frequency of dicentrics decreases quite slowly with time and reliable dosimetry may be performed even months after irradiation. Another advantage of DCA is that the aberrations are detected in peripheral blood lymphocytes and therefore sampling is low-invasive. Also, since lymphocytes circulate throughout the organism, dosimetry is possible even when only a part of the body was irradiated. (Sullivan et al. 2013).

However, the DCA also has its drawbacks, the most serious being the fact that it is time consuming and laborious, and therefore poorly suitable for mass casualty scenarios: To reveal dicentrics, lymphocytes must be induced to division and cultivated for 48 h before scoring can begin (Sullivan et al. 2013). Moreover, the method requires highly trained and experienced personnel and is therefore difficult to automate. This, together with the time-consuming procedures, results in low throughput. In 2010, the total capacity of biological dosimetry laboratories in the European Union for DCA was 1493 samples in the triage mode (50 metaphases per donor) and 187 samples in the full mode (500 metaphases per donor) per week, excluding the time needed for lymphocyte culturing (Wojcik et al. 2010). This would be insufficient in the case of a large radiological accident involving thousands of potentially irradiated subjects. In such a situation, the precision of individual dose estimate is less important. An approximate dose estimation or the identification of subjects exposed above or below a given threshold dose would be sufficient to support physicians in providing optimal medical assistance to victims. New assays allowing for rapid identification of exposed subjects are therefore required. Several candidates for new biological dosimetry methods have been proposed, including premature chromosome condensation assay (PCC) (Lindholm et al. 2010), γ-H2AX foci assay (Rothkamm and Horn 2009), electron paramagnetic resonance (EPR)-based assays (Swartz et al. 2007), and methods based on protein or metabolic biomarkers (Coy et al. 2011; Leszczynski 2014). One of the most encouraging new biodosimetry methods is the analysis of gene expression in blood cells at the mRNA level (Amundson et al. 2004; Badie et al. 2013; Chaudhry et al. 2012; Joiner et al. 2011; Kabacik et al. 2011a; Tucker et al. 2012). Several ionizing radiation-responsive genes have been identified, and different methodological approaches have been proposed involving either microarrays or quantitative PCR (qPCR) (Amundson et al. 2000; Boldt et al. 2012; Dressman et al. 2007; Fachin et al. 2007). Although this new approach to biological dosimetry is promising, a considerable amount of work still has to be done to complete validation of the new transcriptional biomarkers over the range of possible exposure scenarios. These include investigating the response after different types and doses of radiation, while also taking into consideration the different time intervals since exposure, in order to establish a time window in which reliable dosimetry based on gene expression analysis might be performed.

In the present work, we selected a new panel of radiation-responsive genes and we demonstrated that the analysis of expression of the selected genes allowed for the identification of irradiated blood samples even 48 h after exposure.

## Materials and methods

### Blood collection and irradiation

**Fig. 1:**
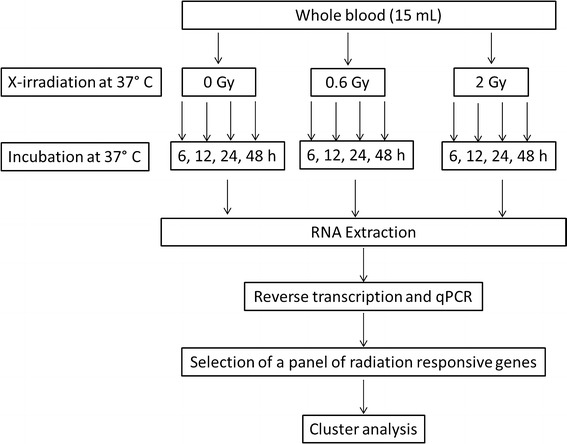
Schematic representation of the experimental procedure

Blood samples were collected from three healthy volunteers (one male, two female) with informed consent from all subjects. A total of 15 mL of blood was collected from each donor in S-Monovette lithium heparin tubes (Sarstedt). Each sample was aliquoted into three tubes (5 mL each) and exposed to 0, 0.6, or 2 Gy of X-rays. X-irradiation was carried out at 37 °C, with the use of a Smart200 (Yxlon) X-ray defectoscope operating at 200 kV and 4.5 mA, with 3-mm Al filtration, at a dose rate of 1.14 Gy/min. Following irradiation, every tube of blood was divided into four tubes (1 mL of the whole blood per tube), one tube for each time point. RPMI 1640 medium (Sigma) supplemented with 10 % fetal calf serum, 100 units/ml penicillin, and 100 µg/ml streptomycin was added to each tube at a 2:1 ratio to the whole blood. The samples were incubated at 37 °C in a humidified incubator with 5 % CO_2_ for either 6, 12, 24 or 48 h. After incubation, the samples were centrifuged at 1600 rcf for 10 min. A two mL portion of the supernatant was discarded, and the rest of the sample was flash-frozen in liquid nitrogen and then stored at −75 °C until RNA extraction. The schematic representation of the experiment is shown in Fig. 1.

### RNA extraction and analysis of gene expression by qPCR

Total RNA was extracted from blood samples using the RiboPure-Blood Kit (Ambion) according to the manufacturer’s protocol including DNase I treatment of the eluted RNA. To assess the concentration and purity of the RNA, a portion of every RNA sample was diluted in TE buffer (pH 8.0) and the absorbance at 230, 260, and 280 nm was measured using Cary 50 UV–Vis spectrophotometer (Varian). All RNA samples used in the subsequent analysis had a concentration over 50 ng/μL, as well as A260/A280 and A260/A230 ratios over 2.0. RNA integrity was tested by agarose gel electrophoresis. Five hundred nanograms of RNA was converted to cDNA in a 20 µL reaction volume using the High-Capacity cDNA Reverse Transcription Kit (Life Technologies) following the manufacturer’s instructions. After completing the reaction, cDNA was diluted to 200 µL with deionized, nuclease-free H_2_O. Subsequently, qPCR was performed in a 20-µL reaction mixture containing 4 µL of the diluted cDNA, 5 µL of deionized, nuclease-free H_2_O, 10 µL of TaqMan Gene Expression Master Mix (Life Technologies), and 1 µL of TaqMan Gene Expression Assay (Life Technologies). The IDs of the TaqMan assays used in the study are given in Table 1. All reactions were run in duplicate. PCR amplification was carried out using a 7500 Real-Time PCR System (Life Technologies) with an initial 10-min denaturation step at 95 °C, followed by 40 cycles of 95 °C for 15 s and 60 °C for 1 min. Relative fold changes in expression were calculated using the ΔΔ*C*_t_ method with *ITFG1* and *DPM1* as reference controls.

**Table 1:** TaqMan gene expression assays used in the study

Gene symbol	Assay ID (Life Technologies)	Context sequence	Amplicon length (bp)
*ACTB*	Hs99999903_m1	TCGCCTTTGCCGATCCGCCGCCCGT	171
*ATF3*	Hs00231069_m1	CACAAAAGCCGAGGTAGCCCCTGAA	108
*BAX*	Hs00180269_m1	CTGGTGCTCAAGGCCCTGTGCACCA	62
*BBC3*	Hs00248075_m1	GAGCGGCGGAGACAAGAGGAGCAGC	101
*BCL2*	Hs00608023_m1	CGGAGGCTGGGATGCCTTTGTGGAA	81
*CDKN1A*	Hs00355782_m1	GCAGACCAGCATGACAGATTTCTAC	66
*DDB2*	Hs03044953_m1	GCCTCTGCAATGGGTTACCACATTC	88
*DPM1*	Hs00187270_m1	AGTTGGGACTAGGAACTGCATATAT	100
*FDXR*	Hs00244586_m1	ACCTGCTAAAGCACCCCCAGGCCCA	71
*GADD45A*	Hs00169255_m1	CGTGCTGGTGACGAATCCACATTCA	123
*GAPDH*	Hs99999905_m1	TTGGGCGCCTGGTCACCAGGGCTGC	122
*GDF15*	Hs00171132_m1	CGCCAGAAGTGCGGCTGGGATCCGG	78
*HPRT1*	Hs01003267_m1	GCAGCCCTGGCGTCGTGATTAGTGA	72
*ITFG1*	Hs00229263_m1	GGAAAATTTGGATGGAAACTTCTCT	102
*MDM2*	Hs00234753_m1	GTGAGGAGCAGGCAAATGTGCAATA	86
*PLK3*	Hs00177725_m1	GAGGAAGAAGACCATCTGTGGCACC	77
*SERPINE1*	Hs01126607_g1	CAACCCCACAGGAACAGTCCTTTTC	71
*SESN2*	Hs00230241_m1	CGTGGAGGAGGTCCTTCGGGAGGGG	65
*TNFRSF10B*	Hs00366278_m1	TCCCACTGAGACTCTGAGACAGTGC	62
*TNFSF4*	Hs00182411_m1	TCTCTGCTCTTCAGGTATCACATCG	72
*VWCE*	Hs00328069_m1	ATCTGCCTGCTGGGCTCAGTGGCCT	94

The “context sequence” is the nucleotide sequence surrounding the region to which the probe binds

### Statistical analysis

A statistical analysis of the obtained data was performed using Statistica 9.0 software (StatSoft). Statistical significance was evaluated by an analysis of variance (ANOVA) followed by a post hoc Tukey’s test. Differences were considered statistically significant when the *p* value was <0.05. Cluster analysis was performed using city-block (Manhattan) distances and the unweighted pair-group method using arithmetic averages.

## Results

### Selection of genes used as reference controls

The experiment was performed as outlined in Fig. 1 and described in the Materials and methods section. Blood from three healthy donors was X-irradiated with a dose of 0, 0.6, or 2 Gy, and RNA was extracted after 6, 12, 24, or 48 h after irradiation. Gene expression was measured by reverse transcription quantitative PCR (RT-qPCR) method in which cDNA is amplified and simultaneously detected using fluorescent dyes or probes. The cycle number at which the fluorescence reaches the defined threshold is called the threshold cycle (*C*_t_) or quantification cycle (*C*_q_) and is used for the quantification of the starting amount of cDNA. To normalize for variation in the amount and quality of RNA between different samples, the expression of a target gene is normalized to one or more reference genes, for which the expression is stable at the given experimental conditions. To this end, the *C*_t_ value of the reference gene is subtracted from the *C*_t_ value of the target gene. The resulting parameter is called Δ*C*_t_ (Schefe et al. 2006).

Among genes, where expression is considered as a suitable reference control for gene expression-based biological dosimetry, the most frequently used are *ACTB*, *GAPDH*, *HPRT1,* and *18S rRNA* (Amundson et al. 2004; Boldt et al. 2012; Budworth et al. 2012; Chauhan et al. 2014; Chi et al. 2013; Fachin et al. 2007; Hyduke et al. 2013; Kabacik et al. 2011a; Manning et al. 2013; Paul and Amundson 2008, 2011; Paul et al. 2013; Riecke et al. 2012; Sudprasert et al. 2006). These genes are also the most often used reference genes in general. Other reference genes were also proposed based on the stability of their expression after irradiation, e.g., *DPM1*, *ITFG1*, *ERP44*, *RPS9* (Filiano et al. 2011; Joiner et al. 2011; Tucker et al. 2012, 2014).

In the present work, we compared the variability of expression of five potential reference genes (*ACTB*, *GAPDH*, *HPRT1*, *DPM1*, and *ITFG1*). Statistical evaluation by ANOVA showed that X-irradiation did not influence the expression of any gene under study (data not shown). Nevertheless, the standard deviations and variances of *C*_t_ values for *ITFG1* and *DPM1* were much lower than for *ACTB*, *GAPDH,* and *HPRT1* (Table 2). Therefore, in the subsequent analyses, the *C*_t_ values of target genes were normalized to the geometric mean of *C*_t_ values for *ITFG1* and *DPM1* according to the following formula:

$$\Delta C_{\text{t}} \left( {\text{target gene}} \right) = C_{\text{t}} \left( {\text{target gene}} \right) - \sqrt {C_{\text{t}} \left( {ITFG1} \right) \times C_{\text{t}} \left( {DPM1} \right)}$$

**Table 2:** Descriptive statistics of *C*_t_ values for the five potential reference genes from 36 samples obtained in the experiment (3 donors × 3 doses × 4 time points)

	Valid N	Mean *C*_t_	Median *C*_t_	Minimum *C*_t_	Maximum *C*_t_	Variance	Std. dev.
*ITFG1* *C*_t_	36	29.94	29.92	28.72	31.72	0.51	0.71
*DPM1* *C*_t_	36	29.17	29.09	27.87	31.25	0.59	0.77
*HPRT1* *C*_t_	36	31.23	30.98	29.71	34.24	0.89	0.95
*GAPDH* *C*_t_	36	25.40	24.97	23.32	28.99	1.59	1.26
*ACTB* *C*_t_	36	23.49	23.45	21.66	26.76	1.84	1.35

### Expression of potential transcriptional biodosimeters

**Fig. 2:**
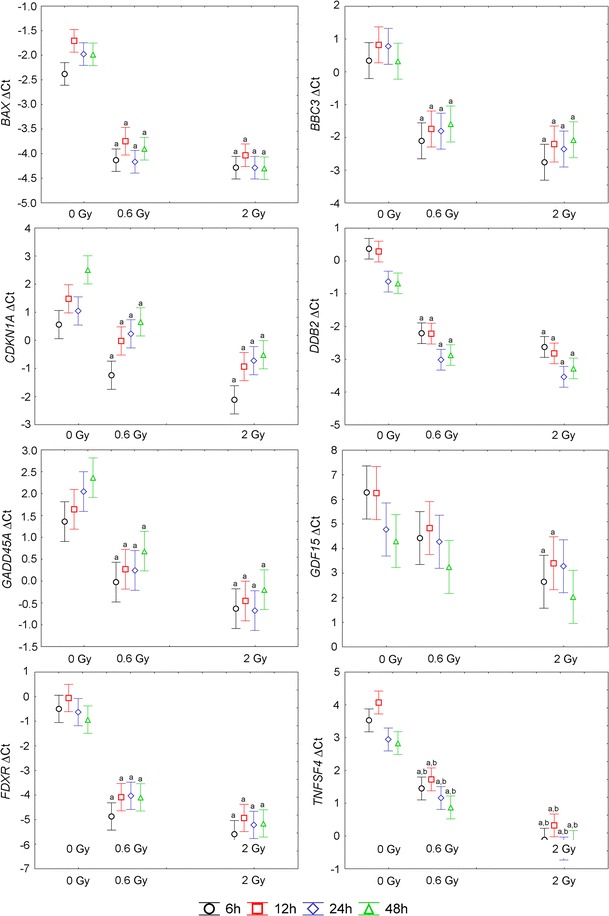
Mean and 0.95 confidence interval of Δ*C*_t_ values for radiation-responsive genes included in the panel. *a* denotes statistically significant difference in post hoc Tukey’s test versus mock-irradiated samples (0 Gy), *b* denotes statistically significant difference in post hoc Tukey’s test between 0.6 and 2 Gy

**Fig. 3:**
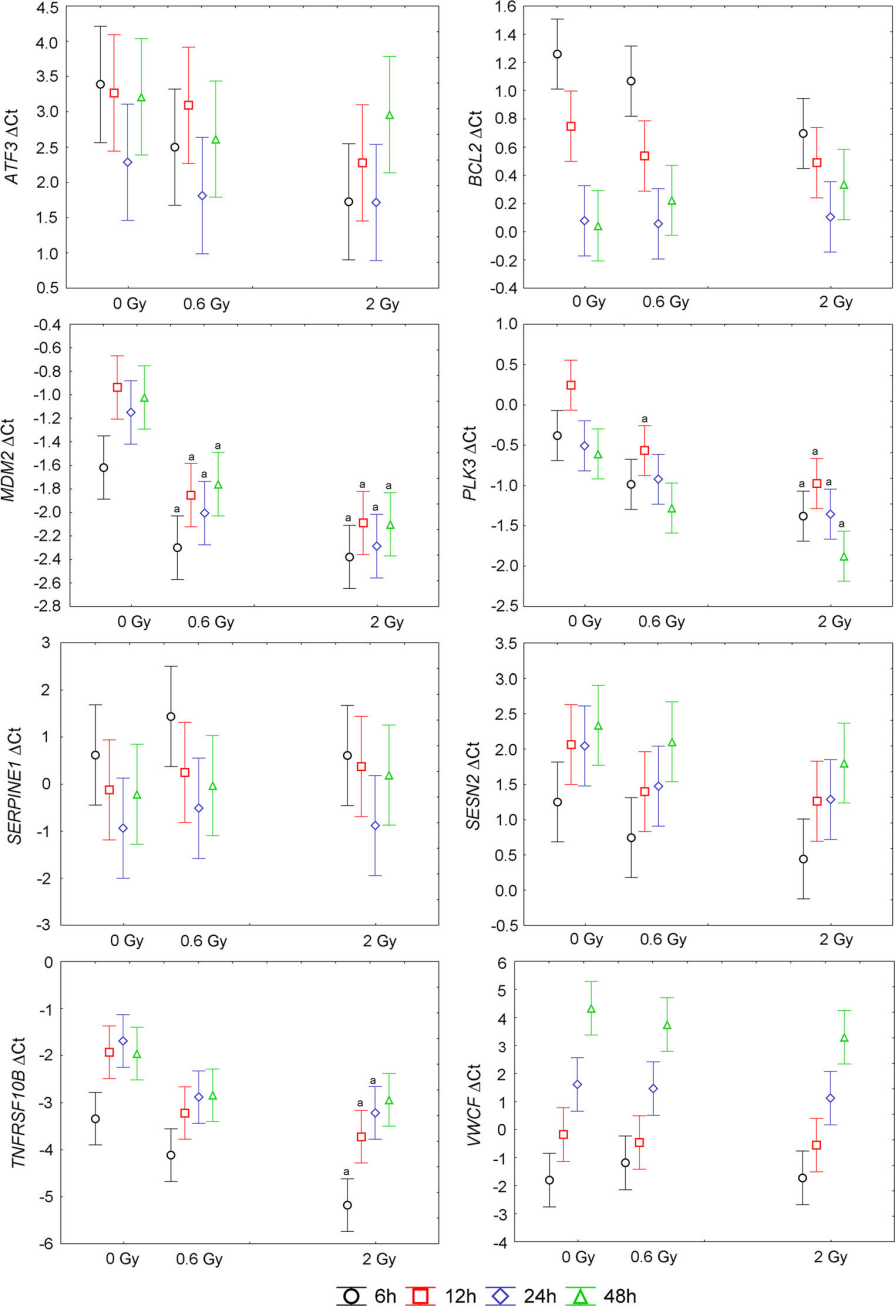
Mean and 0.95 confidence interval of Δ*C*_t_ values for genes not included in the panel. *a* denotes statistically significant difference in post hoc Tukey’s test versus mock-irradiated samples (0 Gy)

We selected 16 genes, the expression of which was previously reported to be up- or down-regulated in blood cells in response to ionizing radiation: *ATF3*, *BAX*, *BBC3*, *BCL2*, *CDKN1A*, *DDB2*, *FDXR*, *GADD45A*, *GDF15*, *MDM2*, *PLK3*, *SERPINE1*, *SESN2*, *TNFRSF10B*, *TNFSF4*, and *VWCE* (Amundson et al. 2003; Boldt et al. 2012; Budworth et al. 2012; Filiano et al. 2011; Grace and Blakely 2007; Kabacik et al. 2011a, b; Li et al. 2011; Riecke et al. 2012; Tucker et al. 2014). Expression of these genes was analyzed by RT-qPCR at 6, 12, 24, and 48 h after X-irradiation with the dose of 0, 0.6, or 2 Gy. The Δ*C*_t_ values for each gene were analyzed by two-way ANOVA with the following factors taken into consideration: radiation dose (three levels: 0, 0.6, 2 Gy), time after irradiation (four levels: 6, 12, 24, 48 h). The detailed results of ANOVA for each gene are presented in Supplementary Table 1. Both factors had a statistically significant impact on mRNA level of the following genes: *GADD45A*, *CDKN1A*, *MDM2*, *SESN2*, *BAX*, *DDB2*, *ATF3*, *PLK3*, *GDF15*, *TNFSF4*, *TNFRSF10B*. The expression of these genes increased with dose and decreased with time. For *BBC3* and *FDXR,* only the radiation dose had a significant impact on their expression, which increased with dose. In the case of *BCL2*, *SERPINE1*, and *VWCE,* only the time factor had a significant impact on the mRNA level (the expression decreased with time). There was no significant interaction between the dose and time factors for any of the genes under study. In Figs. 2 and 3, mean Δ*C*_t_ values for each gene are shown and dose-related differences significant in post hoc Tukey’s test are marked. It is noteworthy that only for *TNFSF4,* a significant difference between doses of 0.6 and 2 Gy has been observed. Mean fold change values for each gene are given in Table 3.

**Table 3:** Mean fold changes in the expression of tested genes in blood cells after X-irradiation

	Mean fold change							
	0.6 Gy				2 Gy			
	6 h	12 h	24 h	48 h	6 h	12 h	24 h	48 h
*SERPINE1*	0.57	0.77	0.75	0.88	1.01	0.71	0.96	0.75
*TNFRSF10B*	1.71	2.45	2.29	1.85	3.58	3.48	2.89	1.98
*VWCE*	0.65	1.22	1.11	1.49	0.94	1.30	1.40	2.04
***GADD45A***	**2.61**	**2.59**	**3.49**	**3.20**	**3.98**	**4.28**	**6.61**	**5.91**
***CDKN1A***	**3.49**	**2.83**	**1.76**	**3.61**	**6.40**	**5.33**	**3.41**	**8.13**
*MDM2*	1.60	1.89	1.81	1.67	1.69	2.22	2.20	2.11
***BBC3***	**5.45**	**5.92**	**6.00**	**3.77**	**8.57**	**8.14**	**8.77**	**5.27**
*SESN2*	1.42	1.59	1.48	1.17	1.75	1.74	1.69	1.45
***BAX***	**3.37**	**4.11**	**4.56**	**3.78**	**3.74**	**5.02**	**4.96**	**4.98**
***DDB2***	**5.97**	**5.67**	**5.22**	**4.56**	**7.99**	**8.62**	**7.50**	**6.05**
*ATF3*	1.85	1.13	1.39	1.51	3.17	1.99	1.48	1.19
*PLK3*	1.52	1.76	1.33	1.59	2.00	2.33	1.80	2.41
***GDF15***	**3.62**	**2.68**	**1.41**	**2.07**	**12.40**	**7.21**	**2.82**	**4.81**
*BCL2*	1.14	1.16	1.02	0.88	1.48	1.20	0.98	0.82
***TNFSF4***	**4.22**	**5.08**	**3.45**	**3.89**	**12.49**	**13.44**	**10.03**	**8.09**
***FDXR***	**20.71**	**16.37**	**10.60**	**8.95**	**34.16**	**29.43**	**24.05**	**18.64**

Genes for which at least twofold changes in expression were observed for all time points for at least one dose are given in bold

For the subsequent analysis, we selected genes for which at least twofold changes in expression were observed for all time points for at least one dose tested. Eight genes met the criteria: *GADD45A*, *CDKN1A*, *BBC3*, *BAX*, *DDB2*, *GDF15*, *TNFSF4*, and *FDXR*.

### Cluster analysis

**Fig. 4:**
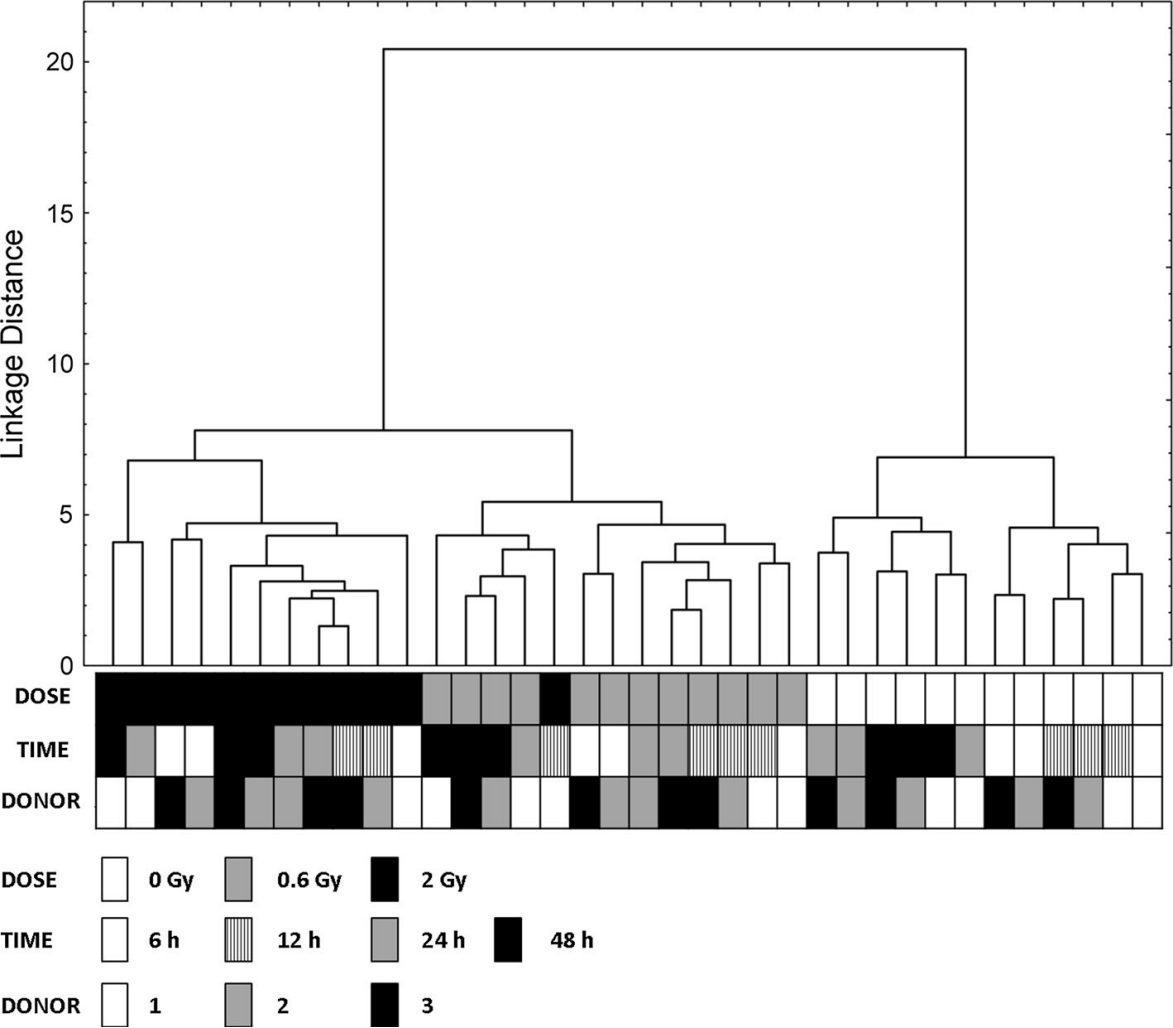
Cluster analysis of 36 blood samples based on Δ*C*_t_ values of *GADD45A*, *CDKN1A*, *BBC3*, *BAX*, *DDB2*, *GDF15*, *TNFSF4*, *FDXR*

To check whether the Δ*C*_t_ values of the selected panel of genes give sufficient information to differentiate between the irradiated and non-irradiated samples and between different doses of radiation, we performed cluster analysis resulting in the tree diagram presented in Fig. 4. In the diagram, two main clusters can be seen, the first one containing the non-irradiated samples and the second one containing the irradiated samples. Inside the first cluster, two smaller clusters were formed, composed of the samples analyzed 6 and 12 h after irradiation or 24 and 48 h after irradiation. The irradiated samples from all time points form a distinct cluster that includes two minor clusters: one consisting of samples irradiated with 2 Gy and the other consisting of samples irradiated with 0.6 Gy (and one misclassified 2 Gy sample). As can be seen, the information from the Δ*C*_t_ values of the selected genes was sufficient to distinguish between the irradiated and non-irradiated samples. The samples were clearly grouped according to the absorbed doses of radiation but not to the time interval since irradiation nor to the blood donor.

## Discussion

In the majority of qPCR experiments, the mRNA level of the target gene is quantified in relation to the mRNA level of one or more reference genes. Thus, the appropriate choice of reference gene(s) is important to minimize variation and obtain reliable results. In our experimental setup, the commonly used reference genes, such as *ACTB*, *GAPDH,* or *HPRT1*, were not the best choice for the analysis of gene expression in blood cells in response to ionizing radiation. Genes *ITFG1* and *DPM1* previously used by Tucker et al. (2012; 2014) and Joiner et al. (2011) showed a lower variability in expression than *ACTB*, *GAPDH,* and *HPRT1* and therefore were used for normalization in our study. A very interesting approach to the problem of normalization of mRNA level was proposed by Forrester and Sprung (2014), who normalized the mRNA level of radiation-modulated transcripts to the level of radiation-independent transcripts from the same gene. Although this innovative approach is potentially very useful, more work is needed to characterize and validate the radiation-dependent and radiation-independent transcripts for each gene of interest.

Many analyses concerning the usefulness of gene expression data for biological dosimetry purposes are based on the fold change in expression between irradiated and non-irradiated samples (Boldt et al. 2012; El-Saghire et al. 2013; Filiano et al. 2011; Kabacik et al. 2011a; Riecke et al. 2012). This is an impractical approach since in the real scenario of a large-scale radiation accident, the data for non-irradiated samples from each donor will not be available and the computing of fold changes will not be possible. To overcome this problem, in our analysis, we used Δ*C*_t_ values, the approach used previously by Tucker et al. (2012, 2013, 2014). Our results confirmed that this approach is correct since the cluster analysis based on Δ*C*_t_ values of selected genes has been able to clearly distinguish between non-irradiated and irradiated samples (Fig. 4). The analysis was not confused by the time that elapsed since irradiation. This indicates that even 48 h after irradiation, the biological dosimetry based on gene expression data may give reliable results. While our analysis was based on in vitro irradiated samples and the conclusions drawn might be limited by the fact that the data from in vitro experiments may not necessarily reflect the conditions of the human body, the data presented by other authors showed that the results of in vitro experiments are in good agreement with the in vivo situation (Amundson et al. 2004; Dressman et al. 2007; Filiano et al. 2011; Paul et al. 2011).

The potential transcriptional biodosimeters analyzed in the present study were chosen based on the literature data. Most of the tested genes were previously shown to be up-regulated in response to ionizing radiation but some of them were reported as down-regulated (*BCL2*, *VWCE*) (Boldt et al. 2012; Grace and Blakely 2007). Not all of the tested genes responded to the ionizing radiation in our experimental setup. This may arise from differences in starting material, experimental protocols or doses, and types of radiation. Among radiation-responsive genes, a significant difference between samples irradiated with 0.6 Gy and 2 Gy was observed only for *TNFSF4*. For the other genes, significant differences were observed between irradiated samples and control samples, but not between samples irradiated with different doses, even though a positive correlation between the dose and mRNA level was observed (Figs. 2, 3). This lack of a sharp difference between samples irradiated with different doses is reflected in the cluster analysis, where one of the samples irradiated with 2 Gy is grouped with 0.6 Gy samples (Fig. 4). This result is in agreement with the data published by other authors, from which it appears that gene expression analysis performs better in distinguishing between irradiated and non-irradiated samples than in predicting the actual absorbed dose (Badie et al. 2013; Filiano et al. 2011; Joiner et al. 2011; Manning et al. 2013; Riecke et al. 2012; Tucker et al. 2013, 2014). This leads to the question concerning the minimal radiation dose necessary to induce gene expression changes marked enough to allow for the identification of irradiated blood samples. Our results showed that this dose is clearly below 0.6 Gy, whereas other authors reported significant changes in gene expression in blood cells at doses as low as 0.02 Gy (Manning et al. 2013) or even 0.005 Gy (Nosel et al. 2013). Further research is needed to define the sensitivity of biodosimetry assay based on gene expression analysis and the minimal absorbed dose that can be detected.

From among the genes significantly up-regulated by irradiation, eight that showed the most pronounced response were selected for further analysis as a panel potentially useful for biological dosimetry purposes. Similar panels were previously tested by other groups and although our panel composition is unique, significant overlapping with other panels is apparent (Badie et al. 2013; Boldt et al. 2012; Joiner et al. 2011; Riecke et al. 2012; Tucker et al. 2014). Our results support the idea that a panel of selected genes may be sufficient for an estimation of the absorbed radiation dose or, at least, for distinguishing between samples exposed above or below a given threshold dose. The latter would be essential in triage after an incident involving exposure to radiation of a large group of people. The analysis of a modest group of genes is far more straightforward and cheaper than the microarray analysis of a large group of transcripts that was also proposed for biological dosimetry purposes (Dressman et al. 2007; Meadows et al. 2008; Paul and Amundson 2008).

Some papers also showed that miRNA level in blood may be affected by ionizing radiation and that it can be used for biological dosimetry (Cha et al. 2009; Chaudhry et al. 2012; Cui et al. 2011; Jacob et al. 2013). This issue was not addressed in the present paper, but we agree that change in miRNA expression might be a potentially useful biodosimetry marker and we plan to perform additional experiments aiming at validation of such miRNA markers and including them in our panel.

The level of transcripts used for biodosimetry purposes may be affected by a variety of confounding factors, such as infections and inflammatory diseases, cigarette smoking, age, sex, genetic polymorphisms. Experiments on mice performed by Tucker et al. (2012) showed that although the expression levels of some genes useful in biological dosimetry are altered by the bacterial endotoxin lipopolysaccharide (LPS), the gene expression analysis may still have utility in biodosimetry even in the presence of a systemic infection. Similar conclusions were drawn by Budworth et al. (2012), who employed the model of human blood irradiated in vitro with the inflammatory stress mimicked by LPS. Certainly, much more research on this issue is needed to validate a reliable and accurate biological dosimeter based on gene expression.

During development of a biological dosimeter based on transcriptional biomarkers, one must consider the type of material to be used for the analysis. RNA could be isolated from the whole blood, total white blood cells (WBC), or their particular type, as well as from plasma or serum if circulating miRNA is to be analyzed. In many papers, gene expression changes after irradiation were analyzed in isolated WBC or lymphocytes (Boldt et al. 2012; Chauhan et al. 2014; Dressman et al. 2007; Fachin et al. 2007; Joiner et al. 2011; Kabacik et al. 2011a; Knops et al. 2012; Meadows et al. 2008; Paul and Amundson 2008; Riecke et al. 2012). This is reasonable, since in the course of their development, erythrocytes lose their nuclei which prevents them from responding to internal and external stimuli by altering gene transcription and mRNA abundance and makes them useless for biological dosimetry. However, the isolation of WBC is possible only from fresh, non-frozen blood. It could be problematic to perform the isolation under field conditions and thus it would require transport of blood samples to the laboratory. During the transport and handling of non-frozen and non-stabilized blood, gene expression in cells would be subjected to change, which could confound the subsequent analysis. Therefore, in the present paper, we successfully tested a different approach. RNA for gene expression analysis was extracted from the whole blood which was frozen immediately after incubation and stored in −75 °C. This approach allows for preservation of gene expression signature at the moment of blood collection. The blood can be then stored and transported in a frozen state to the place where the RNA extraction and gene expression analysis is to be performed. An alternative approach, probably even more practical in field conditions, includes the use of different RNA stabilization reagents that are able to immediately lyse all blood cells and protect RNA from degradation allowing for sample transport and storage at room temperature for several hours or even days (Williams 2010).

 Taken together, in the present study, we have selected and tested a new panel of radiation-responsive genes proving its usefulness for biological dosimetry purposes. Our results confirm that the analysis of expression of a carefully selected group of genes can provide sufficient information to discriminate between irradiated and non-irradiated blood samples. Further research is needed to identify the minimal absorbed radiation dose that can be detected by gene expression analysis and to define the impact of potential confounding factors on the reliability of the transcriptional biomarkers-based biological dosimetry.

## Electronic supplementary material

Supplementary material 1 (PDF 199 kb)

## References

[CR1] Amundson SA, Do KT, Shahab S, Bittner M, Meltzer P, Trent J, Fornace AJ (2000). Identification of potential mRNA biomarkers in peripheral blood lymphocytes for human exposure to ionizing radiation. Radiat Res.

[CR2] Amundson SA, Lee RA, Koch-Paiz CA, Bittner ML, Meltzer P, Trent JM, Fornace AJ (2003). Differential responses of stress genes to low dose-rate gamma irradiation. Mol Cancer Res.

[CR3] Amundson SA, Grace MB, McLeland CB, Epperly MW, Yeager A, Zhan Q, Greenberger JS, Fornace AJ (2004). Human in vivo radiation-induced biomarkers: gene expression changes in radiotherapy patients. Cancer Res.

[CR4] Badie C, Kabacik S, Balagurunathan Y, Bernard N, Brengues M, Faggioni G, Greither R, Lista F, Peinnequin A, Poyot T, Herodin F, Missel A, Terbrueggen B, Zenhausern F, Rothkamm K, Meineke V, Braselmann H, Beinke C, Abend M (2013). Laboratory intercomparison of gene expression assays. Radiat Res.

[CR5] Boldt S, Knops K, Kriehuber R, Wolkenhauer O (2012). A frequency-based gene selection method to identify robust biomarkers for radiation dose prediction. Int J Radiat Biol.

[CR6] Budworth H, Snijders AM, Marchetti F, Mannion B, Bhatnagar S, Kwoh E, Tan Y, Wang SX, Blakely WF, Coleman M, Peterson L, Wyrobek AJ (2012). DNA repair and cell cycle biomarkers of radiation exposure and inflammation stress in human blood. PLoS One.

[CR7] Cha HJ, Shin S, Yoo H, Lee EM, Bae S, Yang KH, Lee SJ, Park IC, Jin YW, An S (2009). Identification of ionizing radiation-responsive microRNAs in the IM9 human B lymphoblastic cell line. Int J Oncol.

[CR8] Chaudhry MA, Omaruddin RA, Kreger B, de Toledo SM, Azzam EI (2012). Micro RNA responses to chronic or acute exposures to low dose ionizing radiation. Mol Biol Rep.

[CR9] Chauhan V, Howland M, Wilkins R (2014). Identification of gene-based responses in human blood cells exposed to alpha particle radiation. BMC Med Genomics.

[CR10] Chi C, Tian R, Liu H, Wang H, Wei J, Guo J, Guo F, Li S (2013). Follow-up study of abnormal biological indicators and gene expression in the peripheral blood of three accidentally exposed persons. J Radiat Res.

[CR11] Coy SL, Cheema AK, Tyburski JB, Laiakis EC, Collins SP, Fornace A (2011). Radiation metabolomics and its potential in biodosimetry. Int J Radiat Biol.

[CR12] Cui W, Ma J, Wang Y, Biswal S (2011). Plasma miRNA as biomarkers for assessment of total-body radiation exposure dosimetry. PLoS One.

[CR13] Dressman HK, Muramoto GG, Chao NJ, Meadows S, Marshall D, Ginsburg GS, Nevins JR, Chute JP (2007). Gene expression signatures that predict radiation exposure in mice and humans. PLoS Med.

[CR14] El-Saghire H, Thierens H, Monsieurs P, Michaux A, Vandevoorde C, Baatout S (2013). Gene set enrichment analysis highlights different gene expression profiles in whole blood samples X-irradiated with low and high doses. Int J Radiat Biol.

[CR15] Fachin AL, Mello SS, Sandrin-Garcia P, Junta CM, Donadi EA, Passos GA, Sakamoto-Hojo ET (2007). Gene expression profiles in human lymphocytes irradiated in vitro with low doses of gamma rays. Radiat Res.

[CR16] Filiano AN, Fathallah-Shaykh HM, Fiveash J, Gage J, Cantor A, Kharbanda S, Johnson MR (2011). Gene expression analysis in radiotherapy patients and C57BL/6 mice as a measure of exposure to ionizing radiation. Radiat Res.

[CR17] Forrester HB, Sprung CN (2014). Intragenic controls utilizing radiation-induced alternative transcript regions improves gene expression biodosimetry. Radiat Res.

[CR18] Grace MB, Blakely WF (2007). Transcription of five p53-and Stat-3-inducible genes after ionizing radiation. Radiat Meas.

[CR19] Hyduke DR, Laiakis EC, Li HH, Fornace AJ (2013). Identifying radiation exposure biomarkers from mouse blood transcriptome. Int J Bioinform Res Appl.

[CR20] Jacob NK, Cooley JV, Yee TN, Jacob J, Alder H, Wickramasinghe P, Maclean KH, Chakravarti A (2013). Identification of sensitive serum microRNA biomarkers for radiation biodosimetry. PLoS One.

[CR21] Joiner MC, Thomas RA, Grever WE, Smolinski JM, Divine GW, Konski AA, Auner GW, Tucker JD (2011). Developing point of care and high-throughput biological assays for determining absorbed radiation dose. Radiother Oncol.

[CR22] Kabacik S, Mackay A, Tamber N, Manning G, Finnon P, Paillier F, Ashworth A, Bouffler S, Badie C (2011). Gene expression following ionising radiation: identification of biomarkers for dose estimation and prediction of individual response. Int J Radiat Biol.

[CR23] Kabacik S, Ortega-Molina A, Efeyan A, Finnon P, Bouffler S, Serrano M, Badie C (2011). A minimally invasive assay for individual assessment of the ATM/CHEK2/p53 pathway activity. Cell Cycle.

[CR24] Knops K, Boldt S, Wolkenhauer O, Kriehuber R (2012). Gene expression in low- and high-dose-irradiated human peripheral blood lymphocytes: possible applications for biodosimetry. Radiat Res.

[CR25] Leszczynski D (2014). Radiation proteomics: a brief overview. Proteomics.

[CR26] Li MJ, Wang WW, Chen SW, Shen Q, Min R (2011). Radiation dose effect of DNA repair-related gene expression in mouse white blood cells. Med Sci Monit.

[CR27] Lindholm C, Stricklin D, Jaworska A, Koivistoinen A, Paile W, Arvidsson E, Deperas-Standylo J, Wojcik A (2010). Premature chromosome condensation (PCC) assay for dose assessment in mass casualty accidents. Radiat Res.

[CR28] Manning G, Kabacik S, Finnon P, Bouffler S, Badie C (2013). High and low dose responses of transcriptional biomarkers in ex vivo X-irradiated human blood. Int J Radiat Biol.

[CR29] Meadows SK, Dressman HK, Muramoto GG, Himburg H, Salter A, Wei Z, Ginsburg GS, Chao NJ, Nevins JR, Chute JP (2008). Gene expression signatures of radiation response are specific, durable and accurate in mice and humans. PLoS One.

[CR30] Nosel I, Vaurijoux A, Barquinero JF, Gruel G (2013). Characterization of gene expression profiles at low and very low doses of ionizing radiation. DNA Repair (Amst).

[CR31] Paul S, Amundson SA (2008). Development of gene expression signatures for practical radiation biodosimetry. Int J Radiat Oncol Biol Phys.

[CR32] Paul S, Amundson SA (2011). Gene expression signatures of radiation exposure in peripheral white blood cells of smokers and non-smokers. Int J Radiat Biol.

[CR33] Paul S, Barker CA, Turner HC, McLane A, Wolden SL, Amundson SA (2011). Prediction of in vivo radiation dose status in radiotherapy patients using ex vivo and in vivo gene expression signatures. Radiat Res.

[CR34] Paul S, Smilenov LB, Amundson SA (2013). Widespread decreased expression of immune function genes in human peripheral blood following radiation exposure. Radiat Res.

[CR35] Pinto MM, Santos NF, Amaral A (2010). Current status of biodosimetry based on standard cytogenetic methods. Radiat Environ Biophys.

[CR36] Riecke A, Rufa CG, Cordes M, Hartmann J, Meineke V, Abend M (2012). Gene expression comparisons performed for biodosimetry purposes on in vitro peripheral blood cellular subsets and irradiated individuals. Radiat Res.

[CR37] Rothkamm K, Horn S (2009). gamma-H2AX as protein biomarker for radiation exposure. Ann Ist Super Sanita.

[CR38] Schefe JH, Lehmann KE, Buschmann IR, Unger T, Funke-Kaiser H (2006). Quantitative real-time RT-PCR data analysis: current concepts and the novel “gene expression’s CT difference” formula. J Mol Med (Berl).

[CR39] Stephan G, Oestreicher U, Romm H, Obe G, Vijayalaxmi (2007). Biological dosimetry. Chromosomal alterations—methods, results and importance in human health.

[CR40] Sudprasert W, Navasumrit P, Ruchirawat M (2006). Effects of low-dose gamma radiation on DNA damage, chromosomal aberration and expression of repair genes in human blood cells. Int J Hyg Environ Health.

[CR41] Sullivan JM, Prasanna PG, Grace MB, Wathen LK, Wallace RL, Koerner JF, Coleman CN (2013). Assessment of biodosimetry methods for a mass-casualty radiological incident: medical response and management considerations. Health Phys.

[CR42] Swartz HM, Burke G, Coey M, Demidenko E, Dong R, Grinberg O, Hilton J, Iwasaki A, Lesniewski P, Kmiec M, Lo KM, Nicolalde RJ, Ruuge A, Sakata Y, Sucheta A, Walczak T, Williams BB, Mitchell C, Romanyukha A, Schauer DA (2007). In vivo EPR For dosimetry. Radiat Meas.

[CR43] Tucker JD, Grever WE, Joiner MC, Konski AA, Thomas RA, Smolinski JM, Divine GW, Auner GW (2012). Gene expression-based detection of radiation exposure in mice after treatment with granulocyte colony-stimulating factor and lipopolysaccharide. Radiat Res.

[CR44] Tucker JD, Divine GW, Grever WE, Thomas RA, Joiner MC, Smolinski JM, Auner GW (2013). Gene expression-based dosimetry by dose and time in mice following acute radiation exposure. PLoS One.

[CR45] Tucker JD, Joiner MC, Thomas RA, Grever WE, Bakhmutsky MV, Chinkhota CN, Smolinski JM, Divine GW, Auner GW (2014). Accurate gene expression-based biodosimetry using a minimal set of human gene transcripts. Int J Radiat Oncol Biol Phys.

[CR46] Williams MA (2010). Stabilizing the code-methods to preserve RNA prove their worth. Biomark Insights.

[CR47] Wojcik A, Lloyd D, Romm H, Roy L (2010). Biological dosimetry for triage of casualties in a large-scale radiological emergency:capacity of the EU member states. Radiat Prot Dosimetry.

