# Supplementary material for: Toward the development of transcriptional biodosimetry for the identification of irradiated individuals and assessment of absorbed radiation dose
Source: Radiat Environ Biophys. 2015 May 14;54(3):353–63. doi: 10.1007/s00411-015-0603-8 (PMC4510913; doi:10.1007/s00411-015-0603-8)
Supplement: Supplementary file 1 — Supplementary material 1 (PDF 199 kb) [file 411_2015_603_MOESM1_ESM.pdf]

Radiation and Environmental Biophysics

Towards the development of transcriptional biodosimetry for the identification of irradiated individuals and assessment of absorbed radiation dose

Kamil Brzóska[1], Marcin Kruszewski[1,2]

[1]Institute of Nuclear Chemistry and Technology, Centre for Radiobiology and Biological Dosimetry, Dorodna 16, 03-195 Warsaw, Poland

[2]University of Information Technology and Management, Faculty of Medicine, Department of Medical Biology and Translational Research, Sucharskiego 2, 35-225 Rzeszów, Poland

Corresponding author:

Kamil Brzóska

Institute of Nuclear Chemistry and Technology, Centre for Radiobiology and Biological Dosimetry

Dorodna 16

03-195 Warsaw, Poland

Tel. +48 22 504 1226

Fax. +48 22 504 1341

E-mail: k.brzoska@ichtj.waw.pl

Supplementary Table 1. ANOVA results of the gene expression analysis performed after 6, 12, 24, or 48 hours in blood X-irradiated with 0, 0.6, or 2 Gy

Gene	Effect	SS	Degr. Of Freedom	MS	F	p
GADD45A	**DOSE**	**34,18**	**2**	**17,09**	**117,8**	**<0,001**
	**TIME**	**2,37**	**3**	**0,79**	**5,5**	**0,005**
	DOSE × TIME	0,58	6	0,1	0,7	0,677
CDKN1A	**DOSE**	**37,18**	**2**	**18,59**	**104,8**	**<0,001**
	**TIME**	**15,21**	**3**	**5,07**	**28,6**	**<0,001**
	DOSE × TIME	1,58	6	0,26	1,5	0,226
MDM2	**DOSE**	**7,034**	**2**	**3,517**	**68,8**	**<0,001**
	**TIME**	**1,344**	**3**	**0,448**	**8,76**	**<0,001**
	DOSE × TIME	0,175	6	0,029	0,57	0,751
BBC3	**DOSE**	**57,73**	**2**	**28,86**	**136,4**	**<0,001**
	TIME	1,19	3	0,4	1,9	0,160
	DOSE × TIME	0,68	6	0,11	0,5	0,776
SESN2	**DOSE**	**3,298**	**2**	**1,649**	**7,3**	**0,003**
	**TIME**	**7,432**	**3**	**2,477**	**10,97**	**<0,001**
	DOSE × TIME	0,175	6	0,029	0,13	0,991
BAX	**DOSE**	**36,43**	**2**	**18,21**	**335,4**	**<0,001**
	**TIME**	**0,57**	**3**	**0,19**	**3,5**	**0,031**
	DOSE × TIME	0,4	6	0,07	1,2	0,322
DDB2	**DOSE**	**57,94**	**2**	**28,97**	**414**	**<0,001**
	**TIME**	**5,88**	**3**	**1,96**	**28**	**<0,001**
	DOSE × TIME	0,26	6	0,04	0,6	0,718
ATF3	**DOSE**	**4,611**	**2**	**2,306**	**4,816**	**0,017**
	**TIME**	**5,63**	**3**	**1,877**	**3,92**	**0,021**
	DOSE × TIME	2,33	6	0,388	0,811	0,572
PLK3	**DOSE**	**7,1**	**2**	**3,55**	**52,37**	**<0,001**
	**TIME**	**3,104**	**3**	**1,035**	**15,26**	**<0,001**
	DOSE × TIME	0,223	6	0,037	0,55	0,767
GDF15	**DOSE**	**39,4**	**2**	**19,7**	**24,07**	**<0,001**
	**TIME**	**13,17**	**3**	**4,39**	**5,36**	**0,006**
	DOSE × TIME	3,78	6	0,63	0,77	0,602

BCL2	DOSE	0,094	2	0,047	1,08	0,356
	**TIME**	**4,763**	**3**	**1,588**	**36,4**	**<0,001**
	DOSE × TIME	0,645	6	0,108	2,47	0,053
TNFSF4	**DOSE**	**71,57**	**2**	**35,78**	**417,2**	**<0,001**
	**TIME**	**4,31**	**3**	**1,44**	**16,7**	**<0,001**
	DOSE × TIME	0,66	6	0,11	1,3	0,304
FDXR	**DOSE**	**147,9**	**2**	**73,93**	**340**	**<0,001**
	TIME	1,8	3	0,61	2,8	0,061
	DOSE × TIME	1,5	6	0,25	1,1	0,369
SERPINE 1	DOSE	1,22	2	0,608	0,761	0,478
	**TIME**	**12,66**	**3**	**4,218**	**5,282**	**0,006**
	DOSE × TIME	1,12	6	0,186	0,233	0,962
TNFRSF10B	**DOSE**	**14,76**	**2**	**7,379**	**33,53**	**<0,001**
	**TIME**	**16,02**	**3**	**5,341**	**24,26**	**<0,001**
	DOSE × TIME	1,16	6	0,193	0,88	0,527
VWCF	DOSE	1,4	2	0,69	1,07	0,36
	**TIME**	**147,1**	**3**	**49,04**	**76,26**	**<0,001**
	DOSE × TIME	1,5	6	0,25	0,4	0,875
